# Retinal Vessel Segmentation Based on B-COSFIRE Filters in Fundus Images

**DOI:** 10.3389/fpubh.2022.914973

**Published:** 2022-09-09

**Authors:** Wenjing Li, Yalong Xiao, Hangyu Hu, Chengzhang Zhu, Han Wang, Zixi Liu, Arun Kumar Sangaiah

**Affiliations:** ^1^Hunan Province People's Hospital (The First Affiliated Hospital of Hunan Normal University), Changsha, China; ^2^The College of Literature and Journalism, Central South University, Changsha, China; ^3^Mobile Health Ministry of Education-China Mobile Joint Laboratory, Changsha, China; ^4^School of Computer and Communication Engineering, Changsha University of Science and Technology, Changsha, China; ^5^School of Computer Science and Engineering, Central South University, Changsha, China; ^6^School of Computing Science and Engineering, Vellore Institute of Technology University, Chennai, Tamil Nadu, India

**Keywords:** retinal vessel segmentation, COSFIRE, postprocess, computer-aided diagnosis, medical image segmentation

## Abstract

Retinal vessel extraction plays an important role in the diagnosis of several medical pathologies, such as diabetic retinopathy and glaucoma. In this article, we propose an efficient method based on a B-COSFIRE filter to tackle two challenging problems in fundus vessel segmentation: (i) difficulties in improving segmentation performance and time efficiency together and (ii) difficulties in distinguishing the thin vessel from the vessel-like noise. In the proposed method, first, we used contrast limited adaptive histogram equalization (CLAHE) for contrast enhancement, then excerpted region of interest (ROI) by thresholding the luminosity plane of the CIELab version of the original RGB image. We employed a set of B-COSFIRE filters to detect vessels and morphological filters to remove noise. Binary thresholding was used for vessel segmentation. Finally, a post-processing method based on connected domains was used to eliminate unconnected non-vessel pixels and to obtain the final vessel image. Based on the binary vessel map obtained, we attempt to evaluate the performance of the proposed algorithm on three publicly available databases (DRIVE, STARE, and CHASEDB1) of manually labeled images. The proposed method requires little processing time (around 12 s for each image) and results in the average accuracy, sensitivity, and specificity of 0.9604, 0.7339, and 0.9847 for the DRIVE database, and 0.9558, 0.8003, and 0.9705 for the STARE database, respectively. The results demonstrate that the proposed method has potential for use in computer-aided diagnosis.

## Introduction

**Figure 1:**
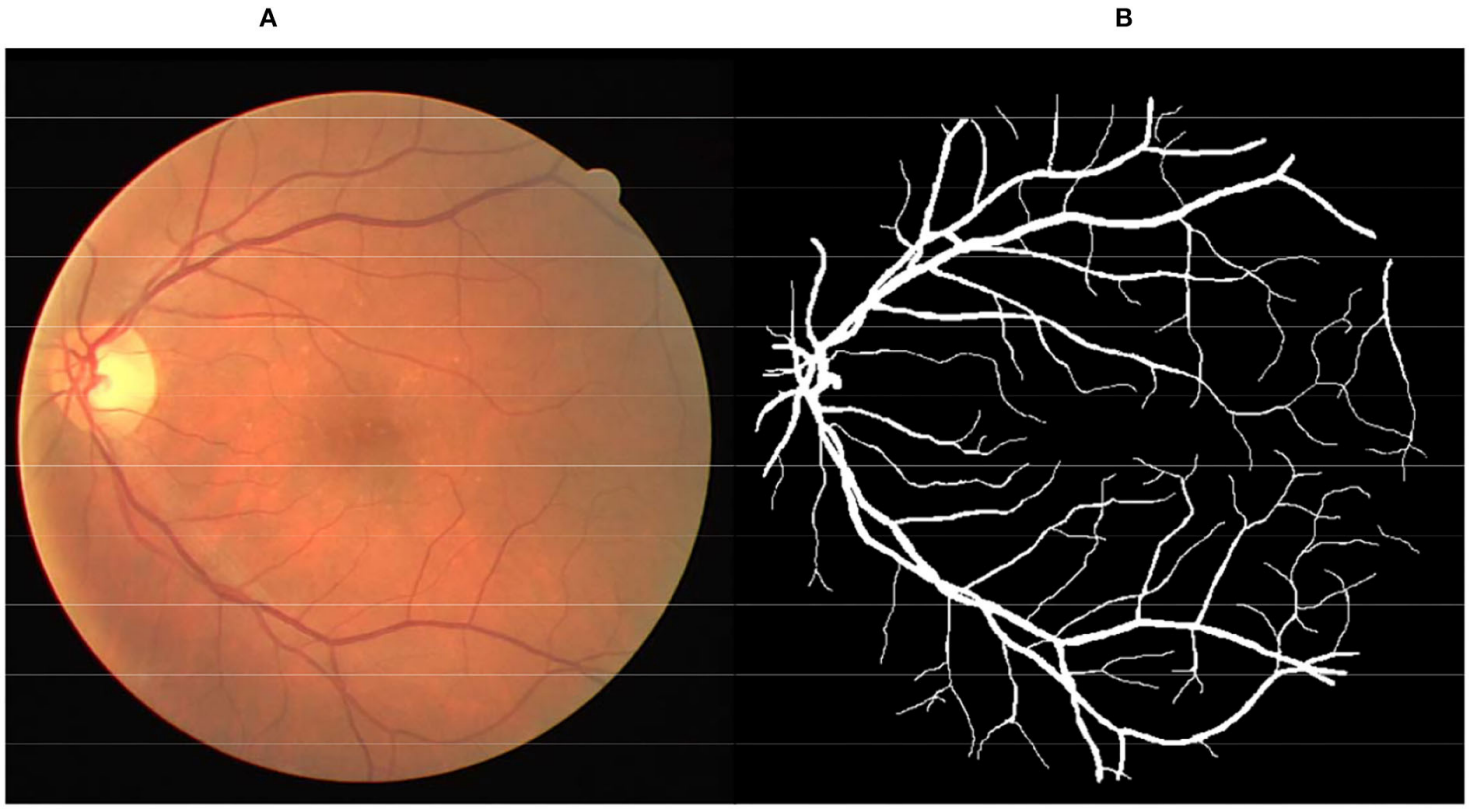
**(A)** Color fundus image from DRIVE database. **(B)** Corresponding manual segmented image.

The color fundus image (Figure 1A) is a non-invasive tool generally used to diagnose various pathologies, including diabetic retinopathy, glaucoma, and age-related macular degeneration. Retinal vessels are the only part of the blood circulation system of humans that can be non-invasively observed directly (1, 2). Inspection of the attributes of the retinal vessel such as width, tortuosity, branching pattern, and angles can play significant roles in early disease diagnosis. Moreover, vessel segmentation is a key and indispensable processing step for further processing operations. This means that the accuracy of vessel segmentation greatly affects the diagnostic effect. But, it is a work of redundancy and boredom for professional ophthalmologists, as shown in Figure 1B. Consequently, to save medical resources and improve the effect of diagnosis, the effective vessel segmentation algorithm with high accuracy and less time consumption is remarkably necessary in computer-aided diagnosis.

A bar-selective COSFIRE filter (B-COSFIRE) is highly efficient for bar-shaped structure detection (3). Therefore, it is a novel and valid method for the automatic segmentation of blood vessels. It is based on the Combination of Receptive Field (CORF) computational model of a simple cell in visual cortex (4) and its implementation, called Combination of Shifted Filter Response (COSFIRE) (5). The COSFIRE filter is trainable, meaning it is not predefined in the implementation, but it is determined from a user-specified prototype pattern (3). COSFIRE has the versatility that can be configured to solve many image processing and pattern matching tasks.

In this article, we propose an efficient retinal vessel segmentation method based on the B-COSFIRE filter based on a previous work mentioned in Azzopardi et al. (3). The original method has good time complexity, but its performance is not as satisfactory as we expected. In the proposed method, we have made many attempts, such as employing denoise operation and post-processing operation. The proposed method has achieved a better performance result without losing the nature of good time complexity.

The rest of this article is organized as follows: In Related Works, we examine the existing blood vessel segmentation method and other applications of COSFIRE filters. In Proposed Methodology, we present our method explicitly. In Experimental Results and Analysis, we introduce the datasets used for experiments and reported the experimental results and the comparisons with existing methods. The discussion for future work and conclusion are presented in Findings and Conclusion, respectively.

## Related Works

Due to the importance of vascular segmentation in computer-aided diagnosis, an efficient vessel segmentation algorithm has always been a research hot spot. Existing approaches for retinal blood vessel segmentation in fundus images can be divided into two groups: unsupervised methods, which include matched filtering, vessel tracking, model-based approaches, and morphological processing, and supervised methods, which use feature vectors to train a binary classification model (6–8). Whether the method is supervised or not depends only on whether manual marking information with a priori is used.

Supervised methods use pixel-wise feature vectors with labeled information, where manually segmented images are referred to as the gold standard, to train a classifier that can distinguish between vascular and non-vascular pixels. This kind of method mainly includes the following two steps: form pixel-wise feature vectors by feature extraction methods and learn a classification model based on vessel and non-vessel training feature vectors (9). Soares et al. (10) reported a segmentation method based on a Bayesian classifier combined with multi-scale analysis for two-dimensional Gabor wavelet transform. Marin et al. (11) proposed a supervised method for retinal vessel segmentation, by applying a multi-layer neural network to classify pixels based on moment-invariant features. Fraz et al. (12) proposed a classification scheme that fuses boosted and bagged decision trees. Aslani et al. (13) presented a supervised method using the random forest classifier, which constructs a rich collection of a 17-dimensional feature vector including B-COSFIRE filter response and trains a random forest classifier to accomplish the segmentation of fundus images. Strisciuglio et al. (14) proposed a method of retinal vessel segmentation by transforming and re-scaling the features composed of the responses of the bank of selected B-COSFIRE filters to train a support vector machine classifier. Zhu et al. (15) presented a supervised ensemble method for segmenting the retinal vessels by extracting a 39-dimensional feature vector to train the extreme learning machine classifier.

Unsupervised methods mainly extract pathological features through linear operation filtering techniques with predefined kernels. Al-Diri et al. (16) proposed an unsupervised method based on the active contour model to achieve retinal vessel measurement and segmentation. Lam et al. (17) presented a multi-concavity modeling approach based on differentiable concavity measure, which can simultaneously process retinal images of two different health states. Azzopardi et al. (3) proposed a method of retinal vessel segmentation based on the COSFIRE approach, called B-COSFIRE filter, by constructing two kinds of the B-COSFIRE filter which are selective for vessel and vessel-ending, respectively. Khan et al. (18) presented an unsupervised method of vasculature segmentation, by applying pixel AND operation between the vessel location map and B-COSFIRE segmentation image. Bahadarkhan et al. (19) proposed a less computational unsupervised automated technique with promising results for the detection of retinal vasculature by using morphological Hessian-based approach and region-based Otsu thresholding. Khan et al. (20) used distinctive preprocessing steps, thresholding techniques, and post-processing steps to enhance and segment the retinal blood vessels. Khan et al. (21) proposed a framework with fast execution and competing outcomes using MISODATA and B-COSFIRE filters to produce better segmentation results.

Generally, supervised methods perform better than other kinds of methods in retinal vessel segmentation, but supervised methods consume a lot of time in the process of classifier training. On the other hand, the advantage of unsupervised learning is that it does not require artificial label information to train the classifier, which makes it more versatile and easier to implement (6, 22). To sum up, a robust method that can have good performance results and does not require much preparation time is necessary for computer-aided diagnosis.

Deep learning has always been a hot topic in the field of computer research. Deep learning can achieve high-quality performance but requires more training time than supervised learning (23, 24). The advantages and disadvantages of deep learning are obvious. Many scholars have attempted using methods of deep learning to segment vessels in retinal images. Melinsca et al. (25) proposed a method using deep neural network to segment retinal vessels. Khalaf et al. (26) proposed a method of retinal vessel segmentation based on convolutional neural networks for deep feature learning. Fu et al. (27) employed deep learning network and fully connected conditional random fields to accomplish retinal vessel segmentation. Deep learning can often achieve better performance than machine learning; however, deep learning requires much training time and better hardware equipment conditions. Yue et al. (28) introduced the multi-scale input layer and dense block to the conventional U-net so that the network can make use of richer spatial context information. Cheng et al. (29) added a dense block to the U-Net network to make each layer's input come from all the previous layer's output, thereby improving the segmentation accuracy of small blood vessels. In order to follow the discussion easily, we summarize the discussed studies in Table 1.

**Table 1:** Related work of retinal segmentation methods.

	**Reference**	**Year**	**Segmentation methods**	**Technology**
Supervised methods	Soares et al. (10)	2006	Combining two-dimensional Gabor wavelet transform multi-scale analysis and Bayesian classifier	2-D Gabor wavelet, bayesian classifier with Gaussian mixtures
Supervised methods	Marin et al. (11)	2011	a multi-layer neural network to classify pixels based on moment-invariant features	A multi-layer feedforward network
Supervised methods	Fra et al. (12)	2012	a classification scheme based on an ensemble of boosted and bagged decision trees	Boosted and bagged decision trees
Supervised methods	Aslani and Sarnel (13)	2016	using the random forest classifier based on a hybrid feature vector for pixel characterization	Random forest classifier, morphological top-hat, B-COSFIRE filter, multi-scale Gabor wavelet
Supervised methods	Strisciuglio et al. (14)	2016	transforming and re-scaling the features composed of the responses of the bank of selected B-COSFIRE filters to train support vector machine classifier	B-COSFIRE filters, SVM classifier, GMLVQ, Genetic algorithm
Supervised methods	Zhu et al. (15)	2016	extracting a 39-dimensional feature vector to train the extreme learning machine classifier	Classification and regression tree (CART)
Unsupervised methods	Al-Diri et al. (16)	2009	An active contour model to achieve retinal vessel measurement and segmentation.	Active contour model, growing algorithm, junction resolution algorithm
Unsupervised methods	Liu and Sun (17)	1993	a multi-concavity modeling approach with differentiable concavity measure	Adaptive tracking algorithm
Unsupervised methods	Azzopardi et al. (3)	2015	proposing B-COSFIRE approach for retinal vessel segmentation	B-COSFIRE filters, CLAHE, Masking
Unsupervised methods	Khan et al. (18)	2016	a vasculature segmentation method by applying pixel AND operation between vessel location map and B-COSFIRE segmentation image	CLAHE, morphological filters, difference image of low pass filter, adaptive thresholding, VLM, B-COSFIRE filters
Unsupervised methods	Bahadarkhan et al. (19)	2016	Using morphological hessian-based approach and region-based Otsu thresholding	CLAHE, morphological filters, Hessian matrix and eigenvalues transformation, Otsu thresholding
Unsupervised methods	Khan et al. (20)	2016	proposing Modified Iterative Self Organizing Data Analysis Technique (MISODATA) for vessel segmentation	CLAHE, MISODATA algorithm, postprocessing
Unsupervised methods	Khan et al. (21)	2020	proposing a framework applicating MISODATA and B-COSFIRE filters with fast execution and competing outcomes.	CLAHE, MISODATA algorithm, B-COSFIRE filters
Unsupervised methods	Ooi et al. (30)	2021	Applying edge detection technology based on Canny algorithm.	CLAHE, Canny algorithm
Deep learning methods	Melinsca et al. (25)	2015	using deep neural network to segment retinal vessels	Deep max-pooling convolutional neural networks
Deep learning methods	Khalaf et al. (26)	2016	Using convolutional neural networks for deep feature learning	Deep Convolutional Neural Networks
Deep learning methods	Fu et al. (27)	2016	employing deep learning network and fully- connected conditional random fields to accomplish retinal vessel segmentation.	FCN, Conditional Random Fields (CRFs)
Deep learning methods	Yue et al. (28)	2019	Improved U-net with Multi-scale input layer and dense block introduced	U-net
Deep learning methods	Cheng et al. (29)	2020	Adding dense block to U-Net network	U-net
Deep learning methods	Li et al. (31)	2021	A scheme based on the combination of U-Net and Dense-Net is proposed	U-net, Dense-net, CLAHE
Deep learning methods	Feng et al. (32)	2022	encoder-decoder structure	Inception, Multiple pyramid pooling modules

**Algorithm 1:** B-COSFIRE vessel segmentation algorithm.

**Input:** Original RGB retinal images
**Output:** Final segmented vessel maps
1*Step*1:
2 Extract green band image G form Original RGB retinal images.
3 Extract CIELab version image L form Original RGB retinal images.
4 *Step*2
5 Using CLAHE algorithm to green band image G.
6 Thresholding the luminosity plane of L and produce the mask M.
7 *Step*3
8 Perform masking operation on G and M.
9 Produce the input I of the following vessel segmentation operation.
10 *Step*4
11 Applicate B-COSFIRE filter on I and produce I1.
12 *Step*5
13 Perform Morphological filters (top-hat) on I1 and produce I2.
14 *Step*6
15 Operating binary threshold on I2 and produce I3.
16 *Step*7
17 Post processing on I3.
18 Return: Final segmented vessel maps

The COSFIRE method was proposed by Azzopardi et al. (5), based on the CORF computational model. Due to the fast and accurate nature of COSFIRE, it has received great attention in the field of image processing and pattern recognition. In addition to the application of retinal vascular segmentation as mentioned before, there are some applications based on the COSFIRE filter in other directions. Azzopardi et al. (33) employed descriptors of different shapes based on trainable COSFIRE filters to recognize handwritten digits, detect vascular bifurcations in segmented retinal images in Azzopardi et al. (34), and achieve gender recognition from face images in Azzopardi et al. (35). Gecer et al. (36) proposed a method that can recognize objects with the same shape but different colors, by configuring different COSFIRE filters in different color channels. Guo et al. (37, 38) further developed the COSFIRE method, by configuring the COSFIRE filter with the inhibition mechanism to recognize architectural and electrical symbols and to detect key points and recognize objects.

The aforementioned work has the following problems:

1. Related supervision methods (10–15) have high computational cost and high time cost in the process of model training. Supervised methods need to use a large number of pixel-level data labels for supervised computation, and it consumes much time to produce segmentation results. As shown in **Tables 6**, **7**, the overhead of the relevant supervised methods in the segmentation time is about 1 min.
2. When considering the combined effects of performance and time overhead, some unsupervised (16–21, 30) methods lack efficient application value. Some unsupervised methods have higher time overhead when obtaining higher segmentation performance. While another part of the unsupervised method achieves lower time overhead, the segmentation performance is also reduced. That is to say, these methods cannot guarantee lower time overhead and a better segmentation effect at the same time; in addition, these methods are not versatile and cannot achieve good results in multiple dataset tests.
3. The images that have been processed by binary thresholding are destroyed by noise. Distinguishing the thin vessel from the vessel-like noise is still a challenge (18, 20, 21).

In this article, an improved unsupervised method based on the B-COSFIRE filter is presented. Compared with traditional methods, the proposed method has stronger robustness. The proposed unsupervised method uses image contrast enhancement algorithms and morphological operations and uses the B-COSFIRE filter, which can effectively extract bar-like blood vessels in the fundus image. While improving the effect, it reduces the time overhead. At the same time, our method uses a post-processing algorithm based on connected domains, which can effectively distinguish small connected blood vessel pixels from vessel-like noise. This proves once again that the proposed method has a great application capability in computer-aided diagnosis.

## Proposed Methodology

**Figure 2:**
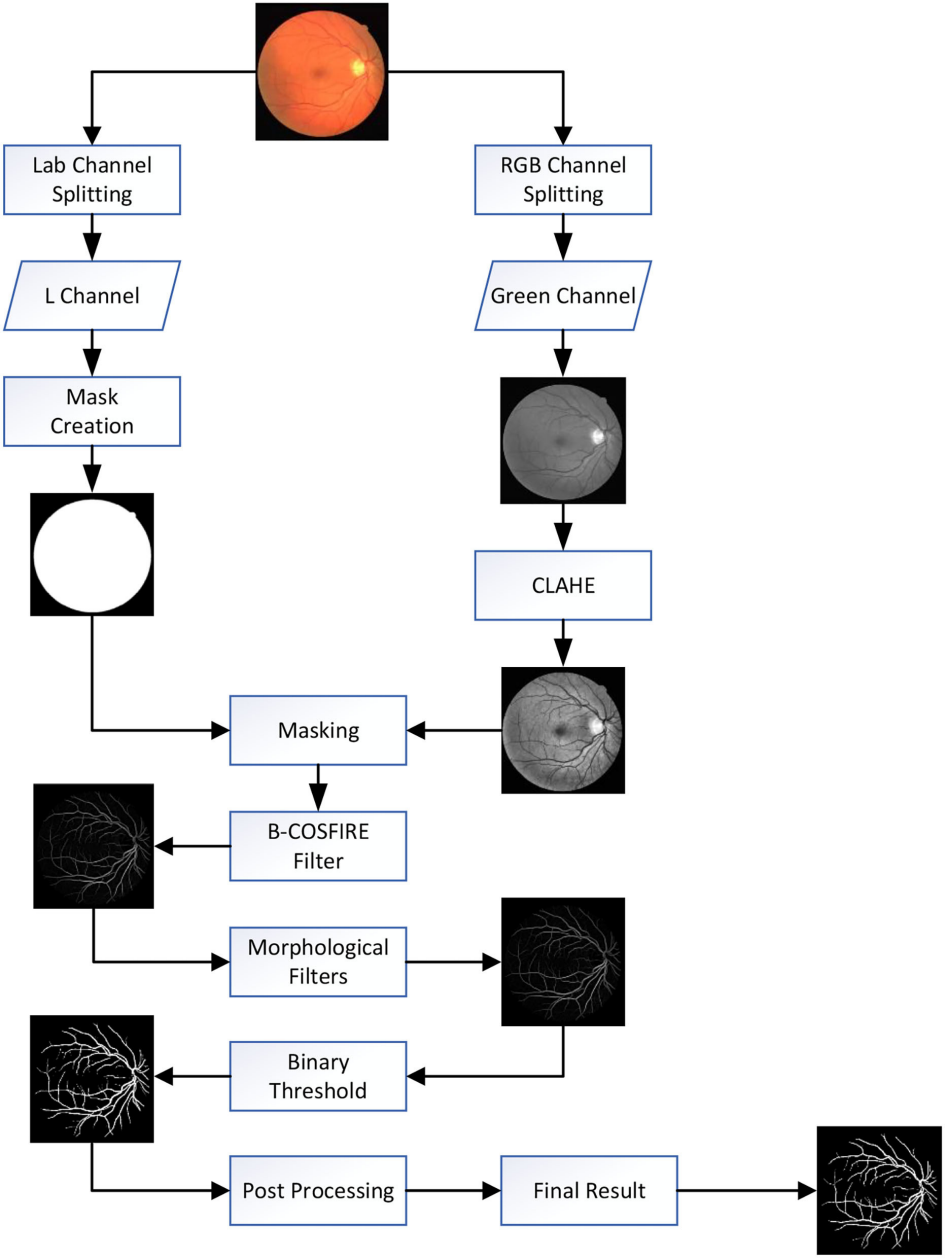
Flowchart of proposed method: first, we using contrast limited adaptive histogram equalization (CLAHE) for contrast enhancement. Second, we threshold the luminance plane of the CIELab version of the original RGB image to produce a mask. Third, we apply the B-COSFIRE filter and morphological filter to detect blood vessels and remove noise. Fourth, the binary threshold was used for vessel segmentation. Finally, unconnected non-vessel pixels are eliminated by post-processing to obtain the final segmentation map.

Vessel segmentation is a basic step in image processing of the fundus image; thus, the process should be fast and efficient. The main idea is taking advantage of the high efficiency of the B-COSFIRE filter and employing other operations that do not need much consumption of time to obtain a better result. Figure 2 represents a flowchart of our proposed method with main processing steps. Algorithm 1 shows the implementation steps of our proposed system. The mission of the B-COSFIRE filter is to select all vessels from the fundus image, while the other operation is to enhance vascular features, denoise background noise, and reduce error classification.

### Preprocessing

Before employing operations to segment vessels, we used the following operations to improve or enhance the characteristics of blood vessels:

a. Green channel extraction: The green band is extracted from RGB retinal images, and the FOV mask (field of view) image is obtained by thresholding the luminosity plane of the CIELab version of the original RGB image. Previous works by Niemeijer et al. (39), Mendonca et al. (40), Soares et al. (10), and Ricci et al. (41) have demonstrated that the green channel of RGB fundus images can best highlight the difference between blood vessels and background, whereas the red and blue channels show low contrast and are very noisy.
b. Mask produce: The FOV mask is an important tool for determining the ROI in the process of vessel segmentation. Although the DRIVE dataset provides the FOV mask, most other datasets do not. For the sake of versatility of the proposed method, we adopt this method to obtain FOV mask images by shifting the original RGB images to CIELab version and then thresholding the luminosity plane.
c. CLAHE: Next, the contrast-limited adaptive histogram equalization (CLAHE) algorithm is used to enhance images for expanding vessels characteristics. The CLAHE algorithm can effectively limit noise amplification in relatively uniform areas and improve local contrast, which is usually used as a preprocessing step in retinal image analysis research (42).

### Segmentation Processing

The proposed segmentation method is based on the feature of the B-COSFIRE filter with the highest correlation to the bar/vessel shape. A B-COSFIRE filter was originally proposed in Azzopardi et al. (3). It takes the responses of a group of difference-of-Gaussians (DoG) filters at certain positions with respect to the center of its area of support as input. In the proposed method, the B-COSFIRE filter has been used for efficiently obtaining vessel-like structures. The B-COSFIRE filter is trainable and is configured to be selective for bar-like structures (3). The term trainable refers to the ability of determining these positions in an automatic configuration process by using a synthetic vessel or vessel-ending image.

The application of the B-COSFIRE filter consists of four main process: Convolution with DoG filters, blurring response, shifting the blurred responses, and estimating a point-wise weighted geometric mean. In the following, we will introduce these steps.

A center-on DoG function with a positive central region and a negative surround denoted by *DoG*_σ_(*x, y*) is given by (3):


(1) $$\begin{array}{l} DoG_{\sigma }\left(x,\;y\right)\overset{\text{def}}{=}\;\frac{1}{2\pi \sigma^{2}}\exp\left(-\frac{x^{2}+y^{2}}{2\sigma^{2}}\right)\\ \;-\;\frac{1}{2\pi {\left(0.5\sigma \right)}^{2}}\exp\left(-\frac{x^{2}+y^{2}}{2{\left(0.5\sigma \right)}^{2}}\right) \end{array}$$

where σ is the standard deviation of the Gaussian function that determines the extent of the surround, 0.5σ is the standard deviation of the inner Gaussian function, and (x, y) represents a pixel location of an image I. The response of a the *DoG* filter *C*_σ_(*x, y*) with a kernel function *DOG*_σ_(*x* − *x′*, *y* − *y′*) is computed by convolution, where (*x′*, *y′*) represents intensity distribution of image I:


(2) $$\begin{array}{l} C_{\sigma }\left(x,\;y\right)\overset{def}{=}|I*DoG_{\sigma }{|}^{+} \end{array}$$

where | ▪ |^+^ half-wave rectification operation is to suppress (set to 0) the negative values.

In the proposed B-COSFIRE filter (3), every point *i* is described by a tuple of three parameters *DoG*(σ_*i*_, ρ_*i*_, ϕ_*i*_), where σ_*i*_ represents the standard deviation of the *DoG* filter that provides the input, ρ_*i*_ and ϕ_*i*_ represent the polar coordinates of the B-COSFIRE filter, and a set of three tuples of a B-COSFRE filter is denoted by *S* = (σ_*i*_, ρ_*i*_, ϕ_*i*_|*i* = 1, .., *n*), where *n* stands for the number of considered *DoG* response.

The blurring operation of the *DoG* filter is shown as follows. It allows for some tolerance in the position of the specific points.


(3) $$\begin{array}{l} \sigma^{\prime }=\sigma_{0}^{\prime }+\alpha \rho_{i} \end{array}$$

where $\sigma_{0}^{\prime }$ and α are constants.

Each blurred *DoG* response is shifted by a distance ρ_*i*_ in the direction opposite to ϕ_*i*_, and they meet at the support center of the B-COSFIRE filter. The blurred and shifted response of a *DoG* filter for each tuple (σ_*i*_, ρ_*i*_, ϕ_*i*_) is denoted by *S*_(_σ__*i*_, ρ_*i*_, ϕ_*i*_)_(*x, y*) in set *S*. The *i*_*th* blurred and shifted *DoG* response is defined as follows:


(4) $$\begin{array}{l} S_{\sigma_{i},\rho_{i},\phi_{i}}(x,\;y)=max_{x^{\prime },y^{\prime }}\{C_{\sigma_{i}}(x-\Delta x_{i}-x^{\prime },y-\Delta y_{i}\\ \;-y^{\prime })G_{\sigma_{i}}(x^{\prime },\;y^{\prime })\} \end{array}$$

where − 3σ′ ≤ *x*′, *y*′ ≤ 3σ′.

Last, the output of the B-COSFIRE filter is defined as the weighted geometric mean of all the blurred and shifted DoG response:


(5) $$\begin{array}{l} r_{S}\left(x,y\right)\overset{def}{=}{\left|{\left({\overset{\left|S\right|}{\underset{i=1}{\prod }}\left(S_{\sigma_{i},\rho_{i},\phi_{i}}\left(x,y\right)\right)}^{\omega_{i}}\right)}^{\frac{1}{\sum_{i=1}^{\left|S\right|}\omega_{i}}}\right|}_{t} \end{array}$$

where $\omega_{i}=exp^{-\frac{\rho_{i}^{2}}{2\sigma^{2}}}$ and |▪|_*t*_ represent thresholding the response at a fraction *t*(0 ≤ *t* ≤ 1). The equation is an AND-type function that a B-COSFIRE filter achieves as response when all *DoG* filter responses are >0.

Moreover, to achieve multi-orientation selectivity, the number of B-COSFIRE filters is configured by using prototype patterns in different orientations. A new set is created by manipulating the parameter ¦ × of each tuple:


(6) $$\begin{array}{l} R_{\psi }\left(S\right)\;=\;\left(\sigma_{i},\;\rho_{i},\;\phi_{i}+\psi \right)|\forall \left(\sigma_{i},\;\rho_{i},\;\phi_{i}\right)\in S \end{array}$$

A rotation-tolerant response is achieved by merging the response of B-COSFIRE filters with different orientation preferences and taking the maximum value at each location (x,y):


(7) $$\begin{array}{l} \overset{\wedge }{r}S\left(x,y\right)\overset{def}{=}\underset{\psi \in \Phi }{\max}r_{R_{\psi }\left(x,y\right)} \end{array}$$

The aforementioned operation is an AND-type function that is achieved by the B-COSFIRE filter when all *DoG* filter responses are non-zero. In total, two kinds of the B-COSFIRE filter are configured (3): symmetric B-COSFIRE filter and asymmetric B-COSFIRE filter. One is selective for vessel, and the other is selective for vessel-ending. For more details, refer to Azzopardi et al. (3).

Morphological filters are used to denoise and to reduce the influence of ophthalmic disorders and to extract useful and meaningful information in small regions of images. Combining image subtraction with openings and closings result in top-hat and bottom-hat transformations (15). The role of the two kinds of transformation is the same, making non-uniform backgrounds uniform and enhancing the image contrast. The top-hat transform is used for light objects on a dark background, so it can make characteristics of vessels more apparent in the dark background. We used the top-hat transform to process the response image produced by the B-COSFIRE filter to enhance vessel structures and reduce noise. As a result, during the threshold segmentation operation, more vessel pixels will be correctly classified and noise pixels will decrease.

The top-hat transformation is defined as follows:


(8) $$\begin{array}{l} G=I-I◦S \end{array}$$

where *I* is a gray-scale image, *S* is a linear structuring element, and ° is opening operation. The top-hat transformation of *I* is defined as *I* minus the opening of *I*. Opening operation can effectively extract useful information of the background that is the same size of the structuring element so that employing top-hat transformation can relatively obtain uniform front-view information.

In this study, the structuring element is square. We experimentally found that the use of morphological top-hat transformation has less noise and better performance in vessel segmentation.

There are two ways to select threshold processing vessel response images. The first method is selecting a manual threshold for each dataset, as in Azzopardi et al. (3). This method does not need too much processing time, but the result is relatively bad. The second one is called adaptive thresholding, which automatically selects the threshold value for each image, instead of the whole dataset, as in Khan et al. (18). In proposed method, we choose the first method to select threshold standing in the point of quickly and effectively segmenting vessels. There are no significant differences between the results obtained by different methods, but the first method can save as much processing time as we expected.

### Post-processing

The images that have been processed by binary thresholding are destroyed by noise. The present results of vessels segmented are far from satisfactory, that is, some vessel pixels are wrongly classified and disappear in the segmentation images. At the same time, many noise points are classified into vessels. So, we consider the post-processing measures to reduce this phenomenon to obtain a better segmentation result.

The adopted post-processing method is based on the connected domain to recover vessels. Specific steps are as follows:

*Step*1. Carry out thinning operation on binary image *I*_*re*_ and then the expansion operation with the template of 3 by 3, followed by morphology complement operation so that 0 surround by 1 in the eight neighborhoods is set to 0.
*Step*2. Repeat *Step* 1 to obtain a relatively complete vascular connected domain and the resulting image *I*_*fin*_.
*Step*3. Obtain *I*_τ_ by taking the intersection of *I*_*re*_ and *I*_*fin*_, then *I*_*fin*_ minus *I*_τ_, and obtain connected regions *I*_*in*_ which can fill *I*_*re*_.
*Step*4. In the model of the eight neighborhoods, in this study, each connected domain of *I*_*in*_ have been used to recover vessels that were denoised. The number of the connected domains before and after the recovering operation have been compared. If reduced, it implies that there are misclassified vascular bifurcations and crossovers in this noise region, and the vascular vessels should be recovered. On the contrary, if it increases, it shows that the noise region is real, and it should not be recovered.
*Step*5. Remove the connected domain that is less than 20 pixels to obtain a better optimized result.

The segmentation results usually consist of some small isolated regions caused by noise, and these regions are sometimes wrongly detected as vessels (18, 20). We used a post-processing method based on connected domains and removed less than or equal to 20 unconnected pixels considered as a non-vessel or a part of the background noise. So the unconnected non-vessel pixel was eliminated, and the thin connected vessel was preserved. After these steps, by identifying and recovering the vascular bifurcations and breakpoints, the continuity and the accuracy of vascular vessels segmentation results were improved. The post-processing operation gives a final resultant binary image.

## Experimental Results and Analysis

In our experiments, the proposed method was evaluated on the publicly available DRIVE database and STARE database. It is worth mentioning that the scarcity of artificial calibration image data is a major obstacle for medical image processing research and development. So, even if the number of images in each database is not big enough, the two databases still occupy a very important position in the field of blood vessel segmentation in fundus images. Since these databases contain ground truth maps manually segmented by different professionals, they have gained popularity in the field of retinal vessel segmentation. Experiments on those two databases also facilitate the comparison of the methods proposed in this article with other methods. Three different measures are used to evaluate the performance. Our experiments were tested with MATLAB in the environment of 2.5 Ghz Intel i5-3210 M CPU and 4 GB memory.

In order to verify the versatility and feasibility of the proposed method, we added a set of experiments, using our method to segment the fundus image in the publicly available CHASEDB1 databases. We used the same three metrics as the aforementioned experiment to evaluate performance. In addition, in order to use a more meaningful measure for the evaluation of the quality of pixel-wise segmentation and to compare with the recent literature to show the effectiveness of our proposed method, the Matthews correlation coefficient (MCC) is introduced (6, 34), the supplementary experiment was tested with MATLAB in the environment of a 2.5 Ghz Intel i5-10300H CPU and 16 GB memory.

### Database

The DRIVE database consists of 40 color images taken by a Canon CR5 3CCD camera with a 45° FOV which are divided into a training set and a test set, each containing 20 images. The size of each image is 768 × 584 pixels, with 8 bits per color channel, and the FOV is circular with 450 pixels in diameter. In the DRIVE database, there is a corresponding mask that delineates the FOV area and the binary vessel segmentation. The images in the test set have been segmented by two human observers, while the images in the training set have been segmented by one observer.

The STARE database consists of 20 color images, and half of the STARE database images contains signs of pathologies. A Topcon TRC-50 fundus camera at 35° FOV was used to acquire the images. The size of each image is 700 × 605 pixels with 8 bits per color channel, and the FOV in the images is around 650 × 550 pixels. The images in the STARE database have been manually segmented by two different observers.

The CHASEDB1 database consists of 28 color images taken from the eyes of 14 school children. Usually, the first 20 images are used for training, and the remaining eight images are used for testing. The size of each image is 999 × 960 pixels, and the binary field-of-view (FOV) mask and segmentation ground truth are obtained by manual methods.

For the comparability of experimental data, the performance of the proposed method is measured on the test set of the DRIVE database and on the all images of the STARE database and CHASEDB1 database by comparing the automatically generated binary images with the ones that are segmented by the first observer as gold standard.

### Evaluation Method

Each resulting binary image was compared with the corresponding gold standard by computing the four performance measurements: true positive (TP) is the number of pixels correctly classified as vessels, false positive (FP) is the number of pixels misclassified as vessels, true negative (TN) is the number of pixels correctly classified as backgrounds, and false negative (FN) is the number of pixels misclassified as backgrounds.

In order to evaluate the performance of our method and compare with state-of-the-art methods, we computed the measures known as accuracy (Acc), sensitivity (Se), and specificity (Sp) in Table 2.

**Table 2:** Performance measures of vessel segmentation.

**Performance measures**	**Description**
Sensitivity(Se)	TP/(TP+FN)
Specificity(Sp)	TN/(TN+FP)
Accuracy(Acc)	(TP+TN)/(TP+FP+TN+FN)

The Acc of one image is a fraction of pixels representing the ratio of the total number of correctly classified pixels to the total number of pixels in the image FOV. Se is determined by dividing the number of pixels correctly classified as vessel pixels by the total vessel pixels in the manual segmentation; thus, Se denotes the ability of correctly identifying the vessel pixels. Sp is determined by dividing the number of pixels correctly classified as background pixels by the total background pixels in the manual segmentation; thus, Sp reflects the ability to detect non-vessel pixels.

In addition, we evaluated MCC indicators referring to Ricci et al. (41). The MCC (7, 21) is a more appropriate indicator of the accuracy of binary categorization in the case of unbalanced structures. The MCC counting transformation is defined as follows:


(9) $$\begin{array}{l} \text{MCC}=\frac{\left(\text{TP}/\text{N}-\text{S}\times \text{P}\right)}{\sqrt{\text{P}\times \text{S}\times \left(1-\text{S}\right)\times \left(1-\text{p}\right)}} \end{array}$$

where N = TN + TP + FN + FP, S = (TP + FN)/N and P = (TP + FP)/N.

### Performance of the Proposed Method

Because the proposed method does not need to train a classifier, images in the training set of the DRIVE database have not been used. We tested on 20 fundus images from the test set of the DRIVE database. The total segmenting time is about 185 s, while each image takes about 9.19 s. The performance results of retinal vessel segmentation on the DRIVE database are shown in Table 3. The average *Acc, Se*, and *Sp* of proposed method are 0.9604, 0.7339, and 0.9847, respectively.

**Table 3:** Performance of proposed method (DRIVE).

	**Time Consuming(s)**	**Se**	**Sp**	**Acc**
Average	9.1852	0.7339	0.9847	0.9604
Maximum	10.1094	0.8455	0.9965	0.9696
Minimum	8.2813	0.5006	0.9737	0.9543

For the STARE database, we tested all 20 fundus images since the proposed method does not have to select images to train a classifier. The total segmenting time is about 242 s, while each image takes about 12.28 s. The performance results of retinal vessel segmentation on the STARE database are shown in Table 4. The average *Acc, Se*, and *Sp* of the proposed method are 0.9558, 0.8003, and 0.9705, respectively.

**Table 4:** Performance of proposed method (STARE).

	**Time consuming(s)**	**Se**	**Sp**	**Acc**
Average	12.2773	0.8003	0.9705	0.9558
Maximum	15.5938	0.9250	0.9900	0.9764
Minimum	10.7118	0.6426	0.9313	0.9245

For the CHASEDB1 database, we tested all 28 fundus images for the same reason that the proposed method does not have to select images to train a classifier. The total segmenting time is about 251 s, while each image takes about 8.96 s. The average Acc, Se, and Sp of the proposed method are 0.9606, 0.6921, and 0.9842, respectively. We still introduced the Matthews correlation coefficient with a score of 0.5886. The performance results of retinal vessels segmentation on the CHASEDB1 database are shown in Table 5.

**Table 5:** Performance of proposed method (CHASEDB1).

	**Time consuming(s)**	**Se**	**Sp**	**Acc**	**MCC**
Average	9.3600	0.5921	0.9842	0.9606	0.5886
Maximum	10.4271	0.7864	0.9959	0.9717	0.75710
Minimum	7.7188	0.4959	0.9313	0.9554	0.3970

The proposed method can generate the segmentation map corresponding to the fundus map in a short time, as shown in Tables 3–5. The processing time for segmenting DRIVE, STARE, and CHASEDB1 fundus map data using our method can reach 8.2318, 10.7188, and 7.7188s, respectively. This shows that the proposed method has real-time feasibility. In addition, our experiments were tested using MATLAB in different environments, and the test results are shown in Tables 3–5, which shows that our experiments are experimentally feasible.

**Figure 3:**
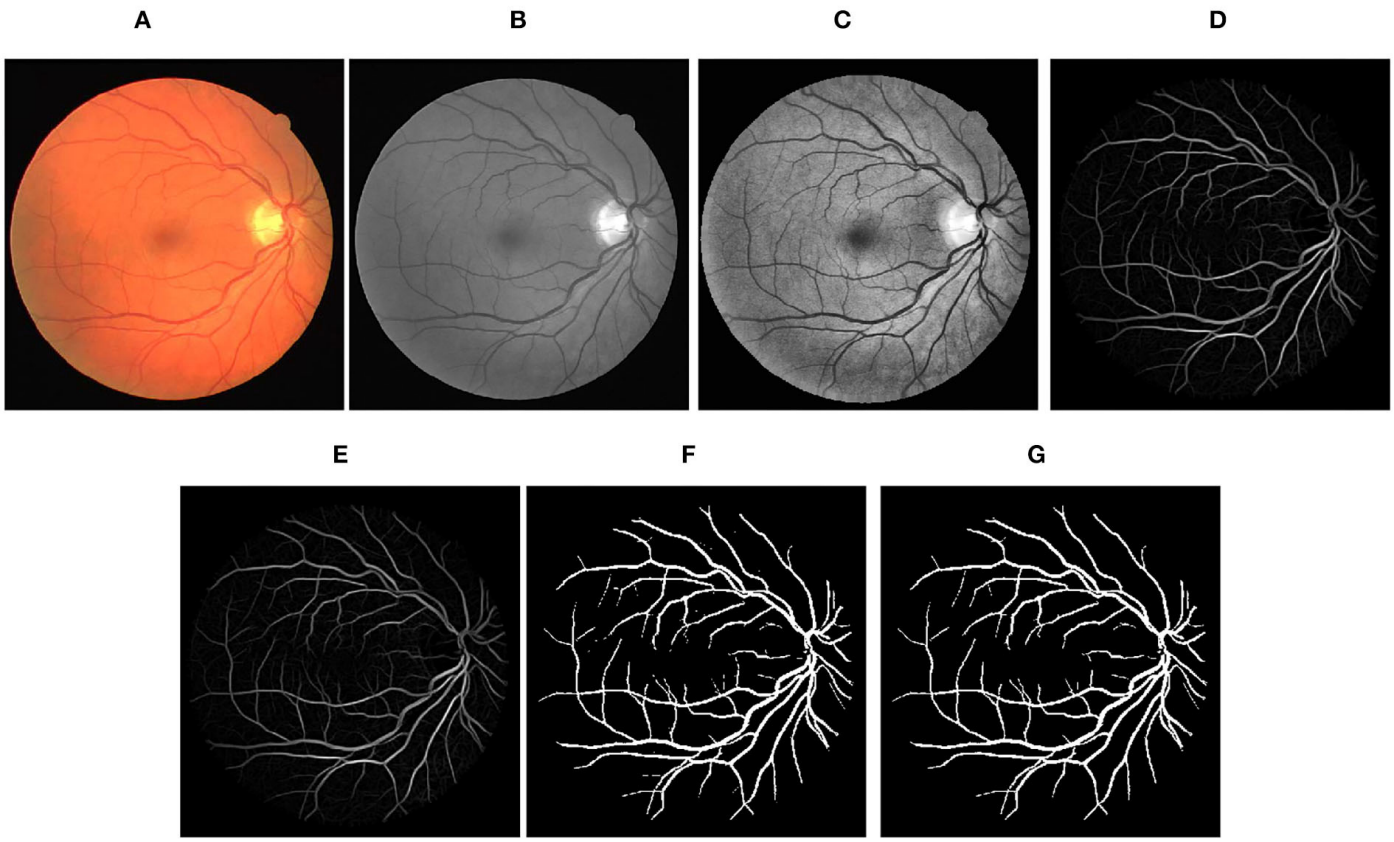
Stepwise illustration of proposed method. **(A)** Color retinal fundus image from DRIVE database. **(B)** Green channel retinal fundus image. **(C)** Processing diagram after CLAHE. **(D)** Processing diagram after B-COSFIRE filters. **(E)** Processing diagram after morphological filters. **(F)** Processing diagram after binary threshold. **(G)** Final segmented image.

**Figure 4:**
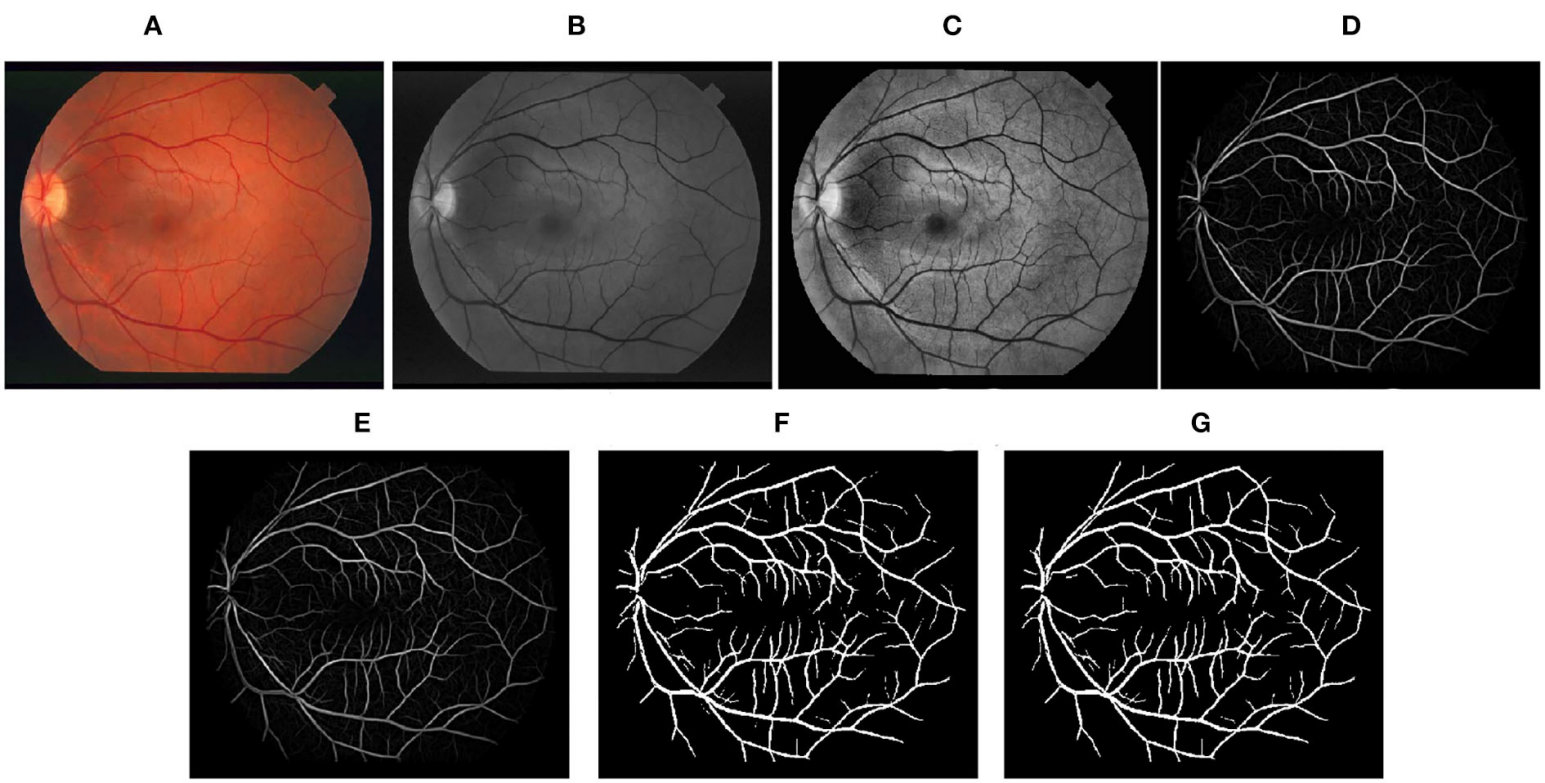
Stepwise illustration of the proposed method. **(A)** Color retinal fundus image from STARE database. **(B)** Green channel retinal fundus image. **(C)** Processing diagram after CLAHE. **(D)** Processing diagram after B-COSFIRE filters. **(E)** Processing diagram after morphological filters. **(F)** Processing diagram after binary threshold. **(G)** Final segmented image.

**Figure 5:**
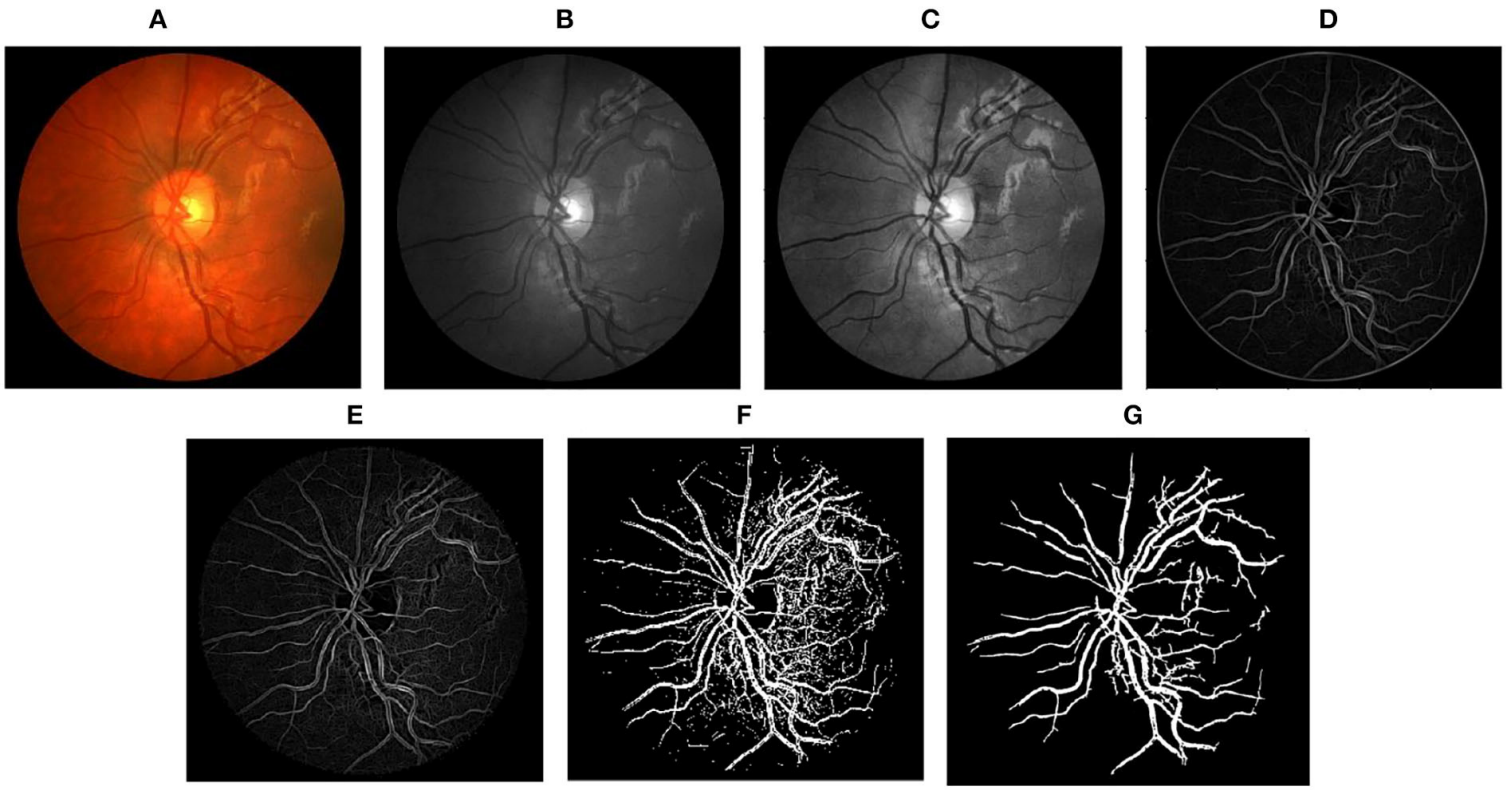
Stepwise illustration of the proposed method. **(A)** Color retinal fundus image from CHASEDB1 database. **(B)** Green channel retinal fundus image. **(C)** Processing diagram after CLAHE. **(D)** Processing diagram after B-COSFIRE filters. **(E)** Processing diagram after morphological filters. **(F)** Processing diagram after binary threshold. **(G)** Final segmented image.

Step-by-step representation of the proposed framework applied to images of DRIVE, STARE, and CHASEDB1 databases has been depicted in Figures 3–5, respectively. In the final segmentation result graph, looking closely at the segmentation of the vessel ends, it is obvious that the method used in this study has good effectiveness in distinguishing between vessel-like noise and thin vessels.

### Comparative Experiment

We compared the proposed B-COSIRE filter approach with other state-of-the-art methods including supervised methods (the top six methods), unsupervised methods (in the middle), and five methods of deep learning (the last four methods) on DRIVE and STARE databases, respectively. The performance in the term of accuracy, sensitivity, and specificity is tabulated in Tables 6, 7, respectively.

**Table 6:** Comparison with other methods (DRIVE).

**Method category**	**Reference**	**Year**	**Segmentation time**	**Se**	**Sp**	**Acc**
Supervised	Soares et al. (10)	2006	3 min	0.7332	0.9782	0.9614
Supervised	Marin et al. (11)	2011	1.5 min	0.7067	0.9801	0.9588
Supervised	Fraz et al. (12)	2012	2 min	0.7406	0.9807	0.9747
Supervised	Aslani and Sarnel (13)	2016	-	0.7545	0.9801	0.9513
Supervised	Strisciuglio et al. (14)	2016	1.5 min	0.7731	0.9708	0.9453
Supervised	Zhu et al. (15)	2016	51 s	0.7462	0.9838	0.9618
Unsupervised	Al-Diri et al. (16)	2009	11 min	0.7282	0.9551	-
Unsupervised	Liu and Sun (17)	2010	13 min	-	-	0.9472
Unsupervised	Azzopardi et al. (3)	2015	10 s	0.7655	0.9704	0.9442
Unsupervised	Khan et al. (18)	2016	10.6 s	0.7155	0.9805	0.9579
Unsupervised	Bahadarkhan et al. (19)	2016	1.56 s	0.776	0.972	0.947
Unsupervised	Khan et al. (20)	2016	-	0.780	0.972	0.952
Unsupervised	Khan et al. (43)	2016	6.1 s	0.747	0.980	0.960
Unsupervised	Khan et al. (21)	2020	5.5 s	0.766	0.972	0.954
Deep learning	Melinsca et al. (25)	2015	-	0.7276	-	0.9466
Deep learning	Khalaf et al. (26)	2016	-	0.8467	0.9494	0.9403
Deep learning	Fu et al. (27)	2016	-	0.7294	-	0.9470
Deep learning	Cheng et al. (29)	2020	-	0.7676	0.9834	0.9559
Unsupervised	Proposed Method	2022	9.19 s	0.7339	0.9847	0.9604

**Table 7:** Comparison with other methods (STARE).

**Method category**	**Reference**	**Year**	**Segmentation time**	**Se**	**Sp**	**Acc**
Supervised	Soares et al. (10)	2006	3 min	0.7207	0.9747	0.9480
Supervised	Marin et al. (11)	2011	1.5 min	0.6944	0.9819	0.9526
Supervised	Fraz et al. (12)	2012	2 min	0.7548	0.9763	0.9534
Supervised	Aslani et al. (13)	2016	-	0.7556	0.9837	0.9789
Supervised	Strisciuglio et al. (14)	2016	2.5 min	0.7668	0.9711	0.9545
Supervised	Zhu et al. (15)	2016	51 s	-	-	-
Unsupervised	Al-Diri et al. (16)	2009	11 min	0.7251	0.9681	-
Unsupervised	Liu and Sun (17)	2010	13 min	-	-	0.9567
Unsupervised	Azzopardi et al. (3)	2015	10 s	0.7716	0.9701	0.9497
Unsupervised	Khan et al. (18)	2016	10.6 s	0.7728	0.9649	0.9518
Unsupervised	Bahadarkhan et al. (19)	2016	1.56 s	0.895	0.939	0.935
Unsupervised	Khan et al. (20)	2016	-	0.745	0.74	0.957
Unsupervised	Khan et al. (43)	2016	6.1 s	0.778	0.966	0.951
Unsupervised	Khan et al. (21)	2020	5.5 s	0.792	0.997	0.996
Deep learning	Melinsca et al. (25)	2015	-	0.7276	-	0.9466
Deep learning	Khalaf et al. (26)	2016	-	0.8467	0.9494	0.9403
Deep learning	Fu et al. (27)	2016	-	0.7294	-	0.9470
Deep learning	Cheng et al. (29)	2020	-	-	-	-
Unsupervised	Proposed method	2022	12.28 s	0.8003	0.9705	0.9558

The proposed framework shows the almost highest results on the DRIVE images for supervised, unsupervised, and deep learning methods, with Acc = 0.9604, Se = 0.7339, and Sp = 0.9847. Our proposed technique also showed high efficiency in terms of sensitivity and specificity among all kinds of techniques on the STARE dataset. The accuracy Acc = 0.9558 also shows a great performance among the methods compared.

In the test experiments on DRIVE and STARE datasets, the proposed method consumed much lower time overhead than supervised methods and deep learning methods and also has advantages over some unsupervised methods. In the CHASEDB1 dataset supplementary experiment, we tested 28 fundus images; the total segmentation time was 251 seconds and the average time was 8.96 s, which shows that our time overhead is relatively low. Furthermore, in the experimental phase, our method does not need to use a large number of image annotations for training. This study used three lightweight public datasets but obtained relatively high test performance, as shown in Tables 6, 7. It can be seen that the proposed method has extremely low overhead on the dataset.

As illustrated before, the proposed unsupervised method is better than other unsupervised methods. The performance of the proposed method is almost the same as that of the supervised methods, although is slightly worse than that of the best performance of deep learning methods. It is important that the proposed method is very efficient, while supervised methods and deep learning methods both consume much time in the process of classifier training. It proves the favorable applicability of the proposed method, which is fast and effective in the field of computer-aided diagnosis.

## Findings

In our work, we proposed a vessel segmentation method of fundus images. In the course of the experiment, we discovered some other issues that may be worth studying. First, in the image preprocessing process, the application of contrast enhancement algorithms to the image will not necessarily lead to better segmentation because some contrast enhancement algorithms will also enhance the background features (44), causing the segmented image to contain more noise. In supplementary experiment, we replaced the CLAHE algorithm in our proposed method with the GLM algorithm referring to Khan et al. (43) and found that the segmentation effect is comparable. We assume this is affected by the environment and data. This is also the direction of our next work. We look forward to making improvements in the method proposed by Khan et al. (43) to further optimize the segmentation method. Second, we found that there are white bars of noise in the blood vessels in part of the binarized images after segmentation. We were able to eliminate it through morphological operations, but the effect still has room for improvement. We believe that we can consider the method of multi-scale input fusion in future.

## Conclusion

This article presents an improved unsupervised method for vascular segmentation on retinal color fundus images. The proposed method is based on the B-COSFIRE filter, through a series of operations including CLAHE, B-COSFIRE filters, morphological filters, and post-process to obtain final binary vessel images. The method is tested on the public databases, DRIVE, STARE, and CHASEDB1. The proposed method requires little processing time (around 9 s for each image in DRIVE and CHASEDB1, 12 s for each image in STARE) and results in the average accuracy, sensitivity, and specificity of 0.9604, 0.7339, and 0.9847 for DRIVE database; 0.9558, 0.8003, and 0.9705 for STARE database; and 0.6921, 0.9842, and 0.9606 for CHASEDB1 database, respectively. In general, the method used in this study has the following advantages: 1. low time overhead and low dataset overhead, 2. good versatility in the field of computer-aided diagnosis, and 3. a relatively high segmentation effect while maintaining a relatively low time overhead. Through the analysis of the experimental results, it is proved that the method proposed in this article is cutting-edge and effective in the field of retinal blood vessel segmentation. In conclusion, the proposed method can be employed for computer-aided diagnosis, disease screening, and any other circumstances that require fast delineation of blood vessels. It may help us prevent many related diseases, such as diabetes and glaucoma. The direction of our future investigation should be done by configuring specific COSFIRE filters of different shapes with other methods of image processing to solve lesion extraction in retinal fundus images.

## Data availability statement

Publicly available datasets were analyzed in this study. This data can be found here: https://blogs.kingston.ac.uk/retinal/chasedb1/; http://www.isi.uu.nl/Research/Databases/DRIVE/download.php.

## Author contributions

WL, YX, and HH are the experimental designers and executors of this study, completed the data analysis, and wrote the first draft of the paper. CZ, HW, and ZL participated in the experimental design and analysis of the experimental results. AS is the project designer and director, who directed the experimental design, data analysis, thesis writing, and revision. All authors read and agree to the final text.

## Funding

This work is supported by the Scientific and Technological Innovation Leading Plan of High-tech Industry of Hunan Province (2020GK2021), the National Natural Science Foundation of China (61702559), the Research on the Application of Multi-modal Artificial Intelligence in Diagnosis and Treatment of Type 2 Diabetes under Grant No. 2020SK50910, the International Science and Technology Innovation Joint Base of Machine Vision and Medical Image Processing in Hunan Province (2021CB1013), and the Natural Science Foundation of Hunan Province (No. 2022JJ30762).

## Conflict of Interest

The authors declare that the research was conducted in the absence of any commercial or financial relationships that could be construed as a potential conflict of interest.

## Publisher's Note

All claims expressed in this article are solely those of the authors and do not necessarily represent those of their affiliated organizations, or those of the publisher, the editors and the reviewers. Any product that may be evaluated in this article, or claim that may be made by its manufacturer, is not guaranteed or endorsed by the publisher.
